# Inhibitory effects of grape skin extract and resveratrol on fatty acid synthase

**DOI:** 10.1186/1472-6882-13-361

**Published:** 2013-12-16

**Authors:** Yan Liang, Weixi Tian, Xiaofeng Ma

**Affiliations:** 1 College of Life Sciences, University of Chinese Academy of Sciences, No. 19A Yuquan Road, Beijing 100049, China; 2 School of Kinesiology and Health, Capital University of Physical Education and Sports, No. 11 Beisanhuanxi Road, Beijing 100191, China

**Keywords:** Fatty acid synthase, β-ketoacyl reductase, Inhibitor, Grape skin extract, Resveratrol, Obesity, 3 T3-L1 Preadipocytes

## Abstract

**Background:**

Grape skin, a rich source of phytochemicals, has been reported to possess remarkable anti-obesity activity. Fatty acid synthase (FAS) is a key enzyme catalyzing the synthesis of fatty acid *de novo*, and has been considered as an anti-obesity target. To elucidate the anti-obesity mechanism of grape skin, we investigated the effects of grape skin extract (GSE) and resveratrol, one of the phytochemicals in GSE, on FAS and FAS over-expressed 3 T3-L1 preadipocyte.

**Methods:**

Purified FAS was obtained from chicken liver. Dried grape skin was extracted by 50% ethanol and partitioned by ethyl acetate. Inhibitory effects of GSE and resveratrol on FAS including fast-binding inhibition, time-dependent inhibition, and enzyme kinetics were determined. Inhibitory effects of GSE and resveratrol on 3T3-L1 preadipocyte were also measured.

**Results:**

GSE inhibited the overall reaction and β-ketoacyl reductase (KR) reaction of FAS with IC_50_ values of 4.61 μg/ml and 20.3 μg/ml. For inhibition by resveratrol, the relevant IC_50_ values were 11.1 μg/ml and 21.9 μg/ml, respectively. And both GSE and resveratrol showed time-dependent inhibition for FAS, with the *k*_obs_ values of 0.028 min^-1^, and 0.040 min^-1^ respectively. They inhibited the overall reaction of FAS competitively with acetyl-CoA, noncompetitively with malonyl-CoA and in a mixed manner with NADPH. Moreover, the inhibition on KR domain by resveratrol was time-dependent with *k*_obs_ value of 0.106 min^-1^. In 3 T3-L1 preadipocytes, resveratrol reduced lipid accumulation remarkably.

**Conclusions:**

GSE and resveratrol are potent FAS inhibitors and they bound reversibly to the KR domain of FAS to inhibit the reduction of the saturated acyl groups in fatty acid synthesis. Based on the valid data and deliberate analysis, we proposed that GSE and resveratrol have great medical potential and officinal value in treating obesity and related diseases.

## Background

Obesity is becoming a worldwide epidemic and is recognized as a worsening factor in a series of chronic diseases [1-3]. It also plays a central role in the metabolic syndrome, which raises the risk of suffering from cardiovascular disease 1.5 to 3 fold [4-6]. Characterized by high body fat mass, obesity is determined by increased adipocyte number (hyperplasia) developing from precursor fat cells or individual fat cell size (hypertrophy) due to triglyceride incorporation [7,8]. Cellular lipid overload or lipotoxicity has been linked to the dysfunction of multiple organs [9-12], and excess of fatty acid ectopic accumulation in these organs will cause lipotoxicity or other obesity-related diseases [13]. The *de novo* synthesis of long chain fatty acids is catalyzed by fatty acid synthase (FAS, EC 2.3.1.85), which has been considered as an anti-obesity target recently [14].

Inside animal bodies, FAS catalyzes the synthesis of long chain saturated fatty acid from the substrates of acetyl-CoA (Ac-CoA), malonyl-CoA (Mal-CoA) and NADPH by its seven functional domains arranged in sequence [15,16]. FAS comprises two identical subunits (260–270 kDa), each of them contains an acyl carrier protein (ACP) and six enzymatic active sites, which are acetyl/malonyl transferase, β-ketoacyl synthase, β-ketoacyl reductase (KR), β-hydroxyacyl dehydratase, enoyl reductase, and thioesterase [15]. FAS is over-transcripted and over-expressed in adipose tissue of genetically-obese rats [17,18] and people with diabetes [19]. It was reported that mice treated with FAS inhibitors led to a reduction of appetite and a dramatic weight loss. The inhibitors restrained the expression of the feeding signal neuropeptide Y, which appeared to be mediated by Mal-CoA, one of the substrates in the FAS catalyzed reaction [14]. Thus, FAS might represent an important link in feeding regulation [14]. In summary, FAS has been considered as a potential therapeutic target for obesity treatment. Its inhibitors, consequently, have favorable application prospects in developing into anti-obesity drugs.

Grape skin extract is a complex mixture of polyphenolics, flavonoids, oligomeric proanthocyanidins, and unsaturated fatty acids that is commonly used as a nutritional supplement. It possessed numerous biological activities and health-promoting properties, such as antioxidant [20], lipid lowering [21], or anti-tumor [22]. Polyphenolic compounds from grape skin have been announced to have many physiological modifications, including anti-obesity [23-25], among which, resveratrol is the most frequently investigated one due to its extensive chemopreventive effects.

Resveratrol (3,5,4′-trihydroxystilbene) is a derivate of stilbene mostly found in grapes and their products, especially red wine [26]. It has the ability to improve the health condition and survival rate of mice on a high-calorie diet [27]. By many measures, mice fed with a high-fat diet plus resveratrol appear as healthy as their lean counterparts, which indicated that resveratrol can protect mice from detrimental effects of diet-induced obesity [27,28]. Resveratrol has been shown to prevent diet-induced obesity and reverse the deleterious effects of obesity including insulin resistance in mice [28]. Moreover, the anti-obesity activity of resveratrol has been corroborated in obese humans in a recent study using low-dose resveratrol supplementation for 30 days [29].

Although have been found anti-obesity function, the effects of the grape skin extract and resveratrol on FAS activity have not been studied comprehensively. Therefore, the aim in the current study was to confirm the inhibitory effects of grape skin extract and resveratrol on FAS and to test their possible inhibitory effects on FAS over-expressed 3 T3-L1 preadipocytes. We demonstrate, for the first time, that the extracts of grape skin and resveratrol potently inhibited the activity of FAS, as well the intracellular lipid accumulation. These results might reveal the health care function of grape and resveratrol from a novel point of view.

## Methods

### Reagents

Ac-CoA, Mal-CoA, NADPH, resveratrol, MTT dye [3-(4, 5-dimethylthiazol-2-yl)-2, 5-diphenyl tetrazolium bromide], 3-isobutyl-1-methylxanthine, insulin, dexamethasone and oil red O were purchased from Sigma-Aldrich (St. Louis, MO, USA). 3 T3-L1 preadipocytes were obtained from the Cell Culture Center of the Institute of Basic Medical Sciences (IBMS), Chinese Academy of Medical Sciences (Beijing, China). Dulbecco’s modified Eagle’s medium (DMEM) and fetal bovine serum (FBS) were purchased from Gibco BRL (Beijing, China). All other reagents were local products with purity of analytical grade. Grape *(Vitis labrusca* L.*)* was purchased from the ChaoShiFa supermarket (Beijing, China) and was identified by Prof. Chuanchu Chen.

### Preparation of grape skin extract

Air-dried grape skin (100 g) was added to 2000 ml of 50% ethanol and extracted for 4 h at room temperature. Grape skin was then removed from the ethanol extract by centrifugation and filtration. The recovered ethanol extracts were evaporated under reduced pressure to yield 25.3 g. A portion (1 g) of the ethanol extracts were suspended in water and partitioned with petroleum ether, ethyl acetate (EtOAc), and n-butanol sequentially to yield four fractions. Among them, EtOAc-soluble fraction (GSE) was chosen and dissolved in DMSO for this study.

### Preparation of FAS and substrates

The FAS used was obtained from chicken liver (Huadu Broiler Corporation, Beijing), since the amino acid sequence of chicken FAS has 63% identity with that of humans [30]. The FAS from chicken liver was purified, stored, and applied as described previously [31]. All animal operations followed the Guidelines for the Care and Use of Laboratory Animals established by the Beijing Association for Laboratory Animal Science, Beijing. The preparation was homogeneous on PAGE in the presence and absence of SDS. The enzyme and substrate concentrations were determined by absorption measurements using the extinction coefficients according to a method previously described [31].

### FAS activity assays

The overall reaction of FAS and β-ketoacyl reduction catalyzed by KR were determined with an Amersham Pharmacia Ultrospec 4300 pro UV–vis spectrophotometer at 37°C by following the decrease of NADPH at 340 nm. The overall reaction mixture contained potassium phosphate buffer, 100 mM, pH 7.0; EDTA, 1 mM; DTT, 1 mM; Ac-CoA, 6 μM; Mal-CoA, 12 μM; NADPH, 37.5 μM and chicken liver FAS 10 μg in a total volume of 2.0 ml [30]. The KR reaction mixture contained ethyl acetoacetate, 40 mM; NADPH, 35 μM; 1 mM EDTA and 15 μg FAS in 100 mM phosphate buffer, pH 7.0, with a total volume of 2.0 ml [32].

### Assay of fast-binding inhibition activity

Fast-binding inhibition was determined by adding the inhibitor into the reaction system before FAS initiated the reaction. This inhibition is generally caused by the non-covalent loading on the enzyme, and is fast and reversible. The final concentration of ethanol did not exceed 0.2% (v/v) in the reaction mixture, so the ethanol did not affect the FAS activity. The extent of inhibition by the addition of inhibitor was measured by reference to the IC_50_ value, which was obtained from a plot of residual activity versus inhibitor concentration.

### Assay of time-dependent inhibition activity

The FAS solution was mixed with inhibitors and incubated at 25°C, and then aliquots were taken to measure the remaining activity at the indicated time intervals to obtain the time course. This time-dependent inhibition is usually caused by a chemical reaction of the inhibitor with the enzyme, and is irreversible. The first-order rate constant of FAS inactivation can be calculated from a semi-log plot of the time course, which is based upon the formula Ln A_t_/A_0_ = - *k*_obs_ t. The A_t_/A_0_ expresses the remaining activity at t time, and *k*_obs_ is the observed first-order rate constant, which is equal to *k*_2_ [I]. The *k*_2_ is the second-order rate constant, which is equal to *k*_obs_/[I] and shows the inhibitory capability. Based on earlier studies [33,34], the inhibition of FAS activity is due to both fast-binding and time-dependent inhibitions, although sometimes the fast-binding reversible inhibition is not potent enough to affect the enzyme.

### Enzyme kinetics study

Possible interference by the inhibitor at each substrate binding site was examined by holding the concentration of the inhibitor at several fixed levels respectively, and increasing one substrate concentration while keeping the concentrations of the other substrates constant. Double reciprocal plots for every concentration of the inhibitors were yielded to estimate the competitive relationship between the variable substrate and inhibitor concentrations. This study is based on fast-binding inhibition.

### Cell culture

3 T3-L1 preadipocytes were cultured in DMEM supplemented with 10% fetal bovine serum at 37°C in the presence of 5% CO_2_. Medium was replaced every 2 days. 3 T3-L1 preadipocytes were seeded in a 24-well plate and grown for 2–4 days for differentiation. Two days after reaching confluence, the medium was changed to DMEM containing 10% FBS supplemented with 0.5 mM 3-isobutyl-1-methylxanthine, 1 μM dexamethasone, and 1.7 μM insulin (day 0). The cells were treated for 2 days (day 2), and then were cultured in DMEM containing 10% FBS and 1.7 μM insulin for another 2 days (day 4). Thereafter, the cells were cultured in DMEM containing 10% fetal bovine serum to day 8, and the medium was changed every 2 days. The resveratrol was added at the beginning of the differentiation process and fresh inhibitor was added whenever a medium change was performed.

### MTT assay

To test the cytotoxicity of resveratrol in 3 T3-L1 preadipocytes, 10 ml of sterile filtered MTT solution (5 mg/ml) in PBS was added to each cell well, reaching a final concentration of 0.5 mg MTT/ml. Unreacted dye was removed after 4 h. The insoluble formazan crystals were dissolved in 200 μl/well DMSO and the absorbance was measured at 490 nm.

### Oil red O staining

Cell differentiation and intracellular lipid accumulation were determined by oil red O staining at day 8 after adipocyte differentiation. The cells were washed twice with phosphate-buffered saline, and stained with 0.3% (w/v) oil red O solution in 60% (v/v) isopropanol for 1 h. After staining, the cells were washed three times with distilled water to remove excess stain. The stained oil droplets in the cells were dissolved in isopropanol, and spectrophotometrically measured at an absorbance of 520 nm.

## Results

### The inhibition of FAS activity by different fractions of grape skin extract

Four fractions (petroleum ether, EtOAc, n-butanol and water) of grape skin were tested to determine their inhibitory activities on FAS. It indicated that GSE showed the highest activity to inhibit FAS with IC_50_ of 4.61 ± 0.4 μg/ml (Table 1). Consequently, GSE was chosen for the further kinetics research.

**Table 1:** The inhibitory activity of the four fractions isolated from grape skin against FAS

**Fractions**	**Mass (mg)**	**FAS inhibitory activity IC**_**50**_**(μg/ml)**
Petroleum ether	15	N/I
Ethyl acetate (GSE)	154	4.61 ± 0.4
n-butanol	161	13.5 ± 1.3
Water	512	16.4 ± 2

N/I indicates that the inhibitory activity can not be detected.

### Inhibition of overall reaction and KR reaction of FAS by GSE and resveratrol

**Figure 1:**
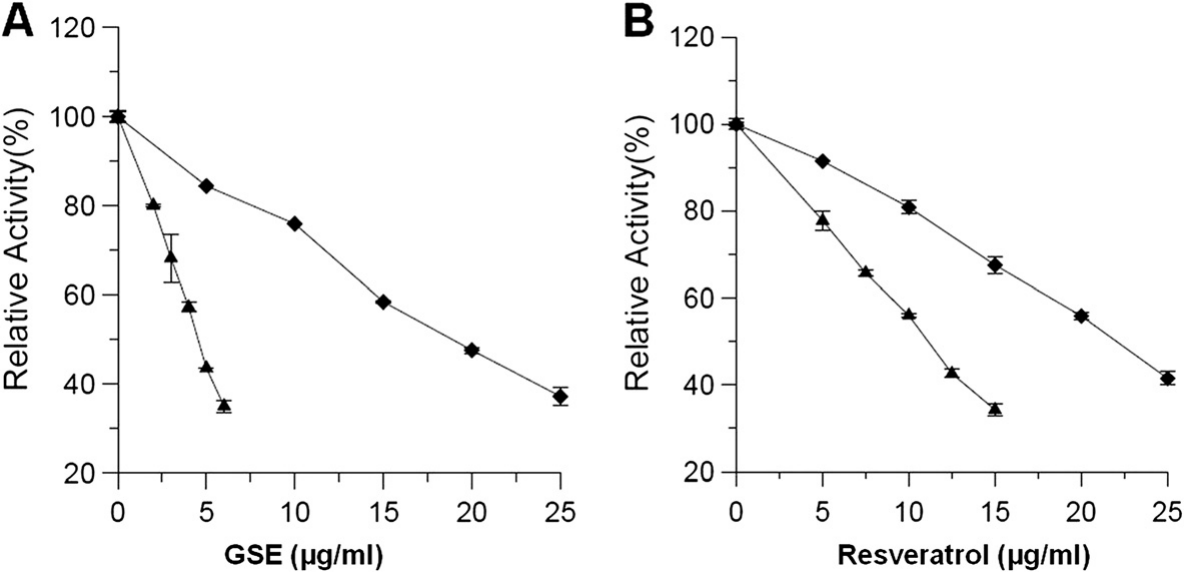
**Fast-binding inhibition by GSE and resveratrol on FAS activities.** The overall reaction (▲) and KR reaction (◆) of FAS were measured in the presence of various concentrations of GSE **(A)** and resveratrol **(B)**. Values represent the mean ± SD of triplicate determinations.

The activities for the FAS overall reaction and KR reaction were assayed to determine the inhibitory capabilities of GSE and resveratrol. By GSE, FAS overall reaction and KR reaction were inhibited with IC_50_ values of 4.61 and 20.3 μg/ml (Figure 1A), while by resveratrol, the relevant IC_50_ values were 11.1 μg/ml and 21.9 μg/ml (Figure 1B).

### Time-dependent inhibition of overall and KR reactions of FAS by GSE and resveratrol

**Figure 2:**
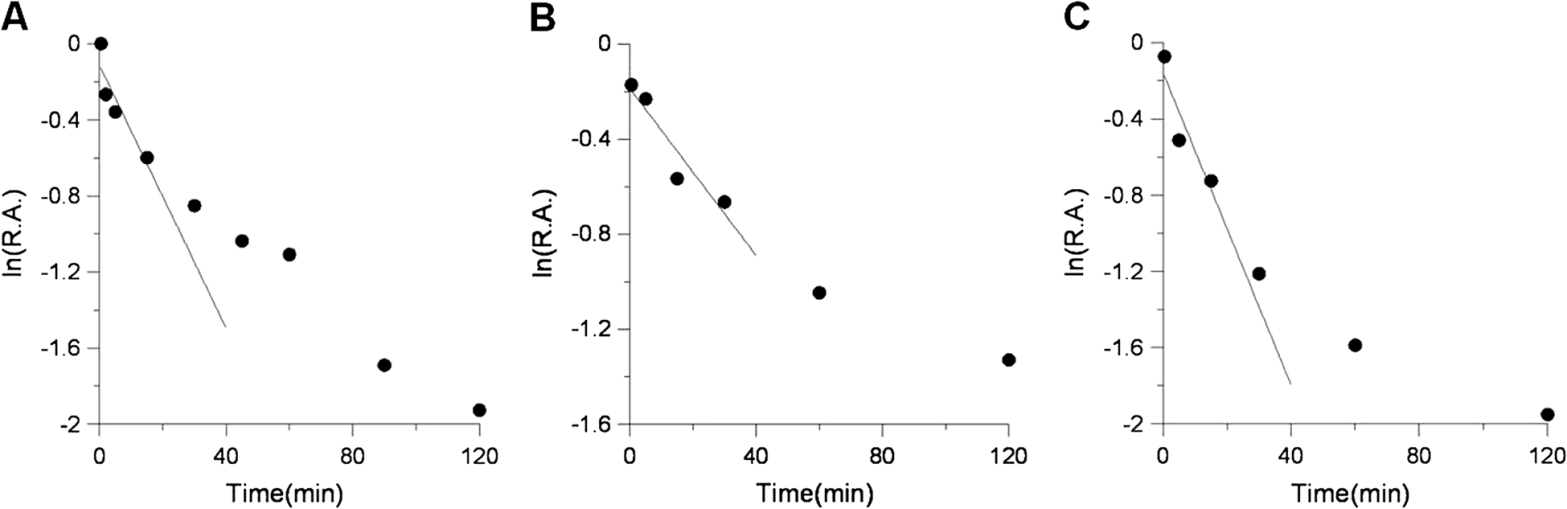
**Time course of time-dependent inhibition by GSE and resveratrol on FAS activities.** The time-dependent inhibition of the overall reaction of FAS was measured in the presence of GSE **(A)** and resveratrol **(B)**. **(C)** The inhibition of KR activity of FAS by resveratrol. The FAS solution was mixed with GSE (3 mg/ml) or resveratrol (3 mg/ml) respectively, and aliquots were taken and assayed for relative activity at the indicated time intervals. (R.A. = relative activity).

Figure 2 showed the time-dependent inhibition processes of FAS overall reactions by GSE (Figure 2A) and resveratrol (Figure 2B), as well the KR reaction inhibited by resveratrol (Figure 2C), respectively. All three reactions underwent a similar time-dependent inhibitory course. FAS lost its activity gradually in 2 h after mixed with GSE or resveratrol, and was totally inactivated by the end of the 4th h (data not shown). The inhibition of all three samples showed two-phase processes, in which the fast phase lasted for about 30 min. Their first-order rate constant (*k*_obs_) obtained from the slope of the semi-log plots of the fast phase were 0.028 min^-1^, 0.040 min^-1^, and 0.106 min^-1^ respectively. The sample concentrations were all 3 mg/ml, and the correspondent *k*_obs_/[I] values, namely the second-order rate constant *k*_2_, were 0.009 (min · mg/ml)^-1^, 0.013 (min · mg/ml)^-1^, and 0.035 (min · mg/ml)^-1^ for these three inhibitions. These results illustrated that resveratrol was one of the effectives in GSE when inhibiting FAS, and it took effect via reacting irreversibly with KR domain. In a word, the time-dependent inhibition of GSE on FAS was mainly due to the reaction between resveratrol and the KR domain.

### Kinetics studies of FAS inhibition by resveratrol

**Figure 3:**
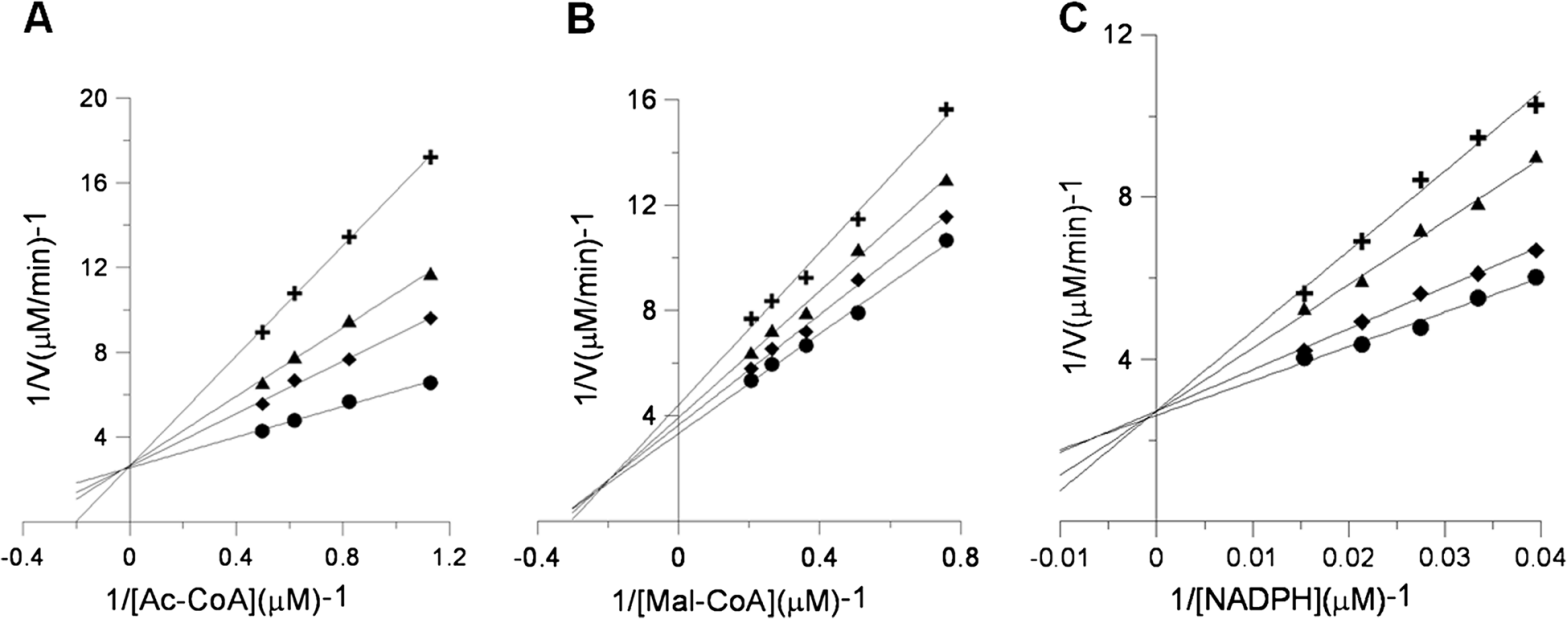
**Lineweaver-Burke plots for inhibition of FAS activity by resveratrol.** Double reciprocal plots for inhibition of FAS by resveratrol. (**A**) The activity of the overall reaction of FAS was measured, and Ac-CoA was the variable substrate. The concentrations of resveratrol were: 0 μM (●), 3.1 μM (◆), 6.2 μM (▲), and 9.3 μM (╋). (**B**) The activity of the overall reaction of FAS was measured, and Mal-CoA was the variable substrate. The concentrations of resveratrol were: 0 μM (●), 2.7 μM (◆), 5.4 μM (▲), and 8.1 μM (╋). (**C**) The activity of KR was measured, and NADPH was the variable substrate. The concentrations of resveratrol were: 0 μM (●), 2.2 μM (◆), 4.4 μM (▲), and 6.6 μM (╋). Each datum is the mean from 2–5 experiments.

The possible interference manner by resveratrol at each substrate-binding site on FAS was kinetically determined. The results of double-reciprocal plots showed that resveratrol inhibited FAS overall activity competitively with respect to Ac-CoA (Figure 3A) and noncompetitively with respect to Mal-CoA (Figure 3B). Consequently, resveratrol possibly bound competitively to the binding site of Ac-CoA or to the binding site of the acetyl moiety from Ac-CoA, to which Mal-CoA or malonyl moiety from Mal-CoA did not bind. Additionally, resveratrol inhibited the KR reaction of FAS competitively with respect to NADPH (Figure 3C), which demonstrated that the binding site of NADPH was also one of resveratrol’s multi-inhibitory targets.

### Effects of resveratrol on 3 T3-L1 preadipocytes

**Figure 4:**
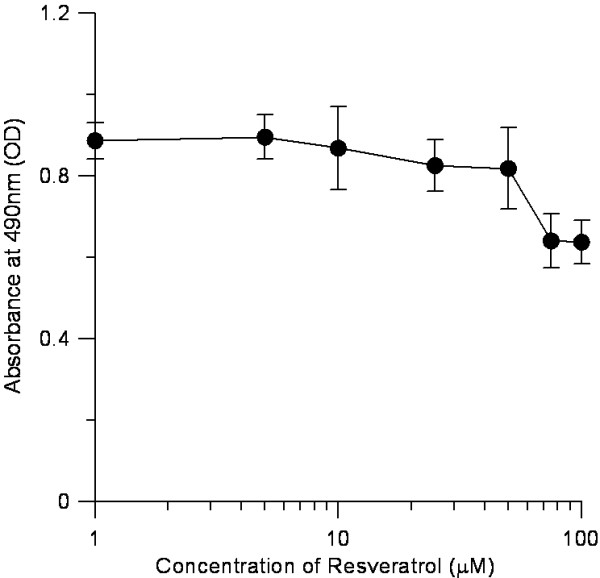
**Cytotoxic effect of resveratrol on 3 T3-L1 preadipocytes.** Cells were treated with 0–100 μM of resveratrol. Intracellular oil droplets were stained with MTT dye and quantified. Cells were incubated with 0–100 μM resveratrol for 24 h at 37°C in humidified 5% CO_2_ incubator. The resultant formazan product was dissolved in 200 ml DMSO/well, and its concentration was measured at 490 nm by a microplate spectrophotometer.

To ensure that the doses of the inhibitors were not generally cytotoxic, 3 T3-L1 preadipocytes were incubated with resveratrol for 48 h, after which a MTT cytotoxicity assay was performed. Resveratrol showed nearly no cytotoxicity to the preadipocytes at doses up to 50 μM, whereas little influence at 75 μM or 100 μM (Figure 4).

**Figure 5:**
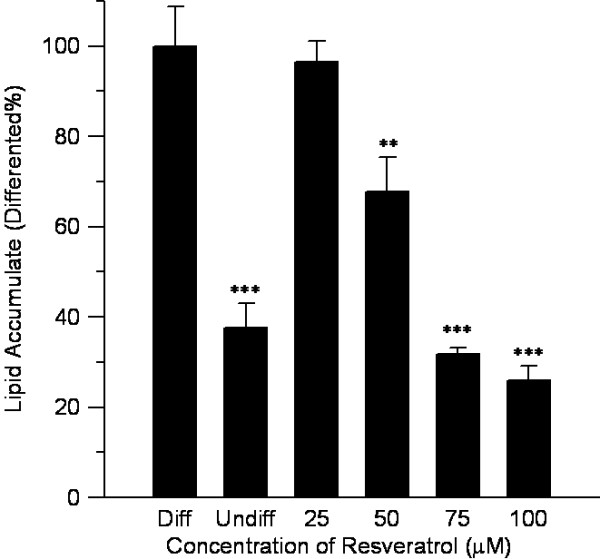
**Inhibitory effects of resveratrol on lipid accumulation.** Stained oil droplets in the cell were dissolved in isopropanol and spectrophoto metrically measured at an absorbance of 520 nm. Resveratrol inhibited 3 T3-L1 intracellular triglyceride accumulation in a dose-dependent manner. n = 4, mean ± SD, Undiff was undifferentiated cell, Diff was differentiated cell, p value obtained using a 2-tailed *T*-test. **p < 0.01, ***p < 0.001 versus the control group.

The results of oil red O staining revealed an obviously reduction of the intracellular triglyceride amount after adding resveratrol into 3 T3-L1 preadipocytes (Figure 5). Resveratrol with concentration of 25, 50, 75, and 100 μM inhibited the cell lipid accumulation to 96.5%, 67.7%, 31.9%, and 26.0% of that of the control differentiated adipocytes, which demonstrated that resveratrol inhibited the differentiation of 3 T3-L1 cell in a dose-dependent manner. In another word, the higher the concentration of resveratrol, the stronger the inhibition of intracellular lipid accumulation it exerted in 3 T3-L1 preadipocytes.

**Figure 6:**
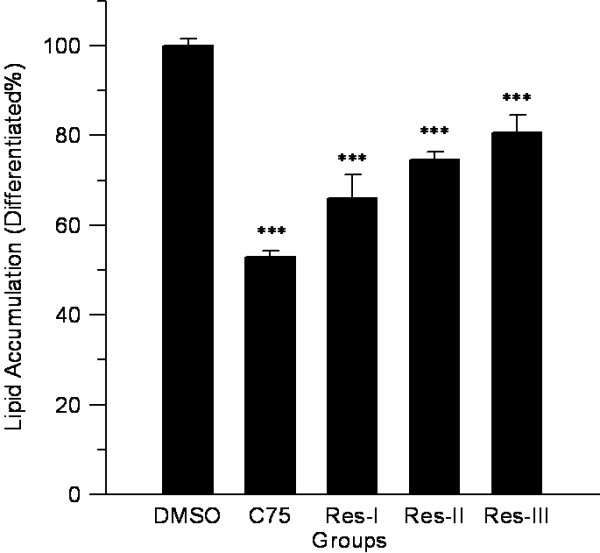
**Effects of resveratrol on lipid accumulation in 3 T3-L1 cells with treatment for different durations.** 3 T3-L1 cells were treated with 50 μM resveratrol for 3 different time intervals: early (Res-I, days 0 to 2), middle (Res-II, days 2 to 4) and late (Res-III, days 4 to 6) stages of differentiation, and intracellular lipid accumulation was measured at an absorbance of 520 nm. DMSO was taken as negative control and C75 as positive control, and the p value was obtained using a 2-tailed *T*-test. ***p < 0.001 versus the control group.

To elucidate the effect of resveratrol during differentiation, 3 T3-L1 cells were treated for 6 days with 50 μM resveratrol for 3 different time intervals. Resveratrol significantly suppressed lipid accumulation up to 34.0%, 25.4%, and 19.3% with treatments during the early, middle, and late stages respectively (Figure 6). These observations suggested that resveratrol affected the signaling for adipocyte differentiation during the early to late cell stages.

## Discussion

In the present work, we have demonstrated that GSE and resveratrol could inhibit the overall reaction and KR reaction of FAS markedly, and the inhibition to the overall reaction included both reversible inhibition and slow-binding inactivation.

GSE and resveratrol are very potent inhibitors of the overall reaction of FAS. Compared with the first reported FAS inhibitor, cerulenin, the IC_50_ values, 4.61 μg/ml of GSE and 11.1 μg/ml of resveratrol, are much lower than that of cerulenin (20 μg/ml) [35]. The strong inhibitory activity of GSE and resveratrol towards FAS, plus their safety, opens up excellent prospects for their application as anti-obesity agents.

We noticed that the inhibitory activity of GSE on FAS was a little stronger than that of resveratrol, thus there must be some other active components in GSE or had combined effect with resveratrol on FAS. So we proposed that, besides resveratrol, GSE as a whole had also a great prospective to be investigated as FAS inhibitors. It was reported in our previous study that teas and tea-polyphenols, which have been considered as functional foods for treating obesity, inhibited FAS activity [32,34,36]. By comparison, GSE and resveratrol exhibited a somewhat stronger inhibitory capability than Longjing green tea (IC_50_ = 12.2 μg/ml) [36], EGCG (epigallocatechin-3-gallate, IC_50_ = 20 μg/ml) [32] and ECG (epicatechin-3-gallate, IC_50_ = 18 μg/ml) [34].

Figure 2 shows the time-dependent inhibition of FAS by both GSE and resveratrol in bi-phase manners with similar rate constants for the fast phase. GSE exhibited higher activity than resveratrol in fast-binding inhibition on the FAS overall reaction, but not on the KR reaction and time-dependent inhibitions. Analyzing these results, it is assumed that GSE contains more flavonoids, which have potent fast-binding inhibition of the FAS overall reaction without inhibition of KR, and which have time-dependent inhibition of FAS [37]. Therefore, the time-dependent inhibition by GSE, which mainly occurs in the KR domain, results from resveratrol. In general, this time-dependent inhibition would express lasting and irreversible activity, thus is important for health preservation. So the inhibitors that exert time-dependent inhibition to FAS, such as resveratrol, C75 and EGCG, have an obvious advantage considering about the prospective *in vivo* application.

Unlike C75 but allied with EGCG, the KR domain is a major reaction site on FAS by the irreversible time-dependent inhibition of resveratrol. However, the resveratrol molecule does not contain a carboxyl group of gallated ester, which has been identified as an active group of EGCG in irreversible time-dependent inhibition [34]. Therefore, the modification mechanism of resveratrol on FAS differs from that of both C75 and EGCG.

As the major FAS inhibitor in grape skin, resveratrol mainly reacts with the KR domain on FAS. The kinetic study of resveratrol suggested that it exerted its inhibition on the site where NADPH binds to the KR domain. This is similar to EGCG [33], which shares some similarities with resveratrol in structure-both of them have two aromatic rings with a certain space and hydroxyl substituents. Moreover, with a smaller molecular structure, resveratrol has a weaker space steric, which may be the reason for its somewhat stronger inhibition activity of FAS.

It is worth noting that extracts of grape flesh expressed no inhibition to FAS (data not shown). This result is consistent with the finding that resveratrol is distributed mainly in grape skin but is scarce in grape flesh. It is also further demonstrated that grape skin plays an important role in the curative effect of grapes, which should not be neglected in nutritional planning and in the processing of grape products. Taking flavor and customs into account, it will be quite acceptable for grape skins to be processed into more food forms without losing its beneficial effects, as exemplified by red wine.

Previous studies on cell lines, animal models, and human epidemiological trials have certified the potential of dietary polyphenols as anti-proliferation agents [38,39]. Polyphenols have also been proven to affect cell growth, differentiation, and apoptosis [40-42]. Our investigation showed that resveratrol effectively depressed the differentiation of 3 T3-L1 preadipocytes. It obviously reduced the size of individual cells that rely on the accumulation of triglyceride in the process from preadipocyte into adipocyte. Resveratrol also inhibited cell population growth of adipocytes in a dose-dependent manner during their whole life cycle. With the reported theory that FAS-generated signals may be essential to support the differentiation of 3 T3-L1 preadipocytes [43], we proposed that resveratrol exhibited inhibition against 3 T3-L1 preadipocytes due to its feature of inhibiting the activity of FAS.

Fat in the animal bodies comes from two main sources: absorption from food and *in vivo* synthesis. High fat in the diet depresses the expression of FAS, and decreases fat synthesis, while excessive absorption of sugar accelerates the expression of FAS [44]. In contrast, Loftus reported that the inhibition of FAS leads to the down-regulation of neuropeptide Y in the hypothalamus, causing a reduction in food intake, which appears to be mediated by Mal-CoA [14]. Furthermore, although the reported inhibitors of FAS, such as C75, cerulenine, EGCG, and resveratrol, have distinct structures, chemical properties, inhibitory mechanisms, and reaction sites on FAS, they all exhibit common effects: decreased food intake, reduced body weight, and inhibited lipid accumulation in adipocytes [14,45,46]. These demonstrate that FAS may play an important role in the regulation of energy metabolism. Once FAS is inhibited, the three substrates of it (Mal-CoA, Ac-CoA, and NADPH) are accumulated. Mal-CoA has been suggested to be a signal molecule in energy metabolism [14,47]. Ac-CoA is the primer of the citric cycle for energy production. NADPH is an important coenzyme with high energy, and the increase of the NADPH/NADP^+^ ratio could help organisms obtain more energy. Therefore, inhibition of FAS leads to the control of the ingestion of energy, the reduction of endogenetic fat, and the promotion of *in vivo* energy production. Consequently, as effective FAS inhibitors, GSE and resveratrol have great potential for clinical treatment of obesity.

## Conclusion

In conclusion, GSE and resveratrol could inhibit FAS activity in both reversible and irreversible manner. Kinetic results confirmed that the main active domain that GSE and resveratrol acted was KR. Since grape and resveratrol are reported to have the ability of treating obesity, we now speculate that they possibly perform, at least in part, by affecting FAS activity.

## Abbreviations

Ac-CoA: Acetyl-CoA; EtOAc: Ethyl acetate; FAS: Fatty acid synthase; GSE: Ethyl acetate extract of grape skin; IC50: The half inhibition concentration; KR: β-ketoacyl reductase; Mal-CoA: malonyl-CoA.

## Competing interests

The authors declare that they have no competing interest.

## Authors’ contributions

WT, as the principal investigator, was responsible for the concept and design of the study. XM conducted the research and wrote the manuscript. YL did the whole experiments of the study. All authors participated in the preparation of, and have approved the final version of the manuscript.

## Pre-publication history

The pre-publication history for this paper can be accessed here:

http://www.biomedcentral.com/1472-6882/13/361/prepub
